# Characterisation of P2Y receptor subtypes mediating vasodilation and vasoconstriction of rat pulmonary artery using selective antagonists

**DOI:** 10.1007/s11302-022-09895-x

**Published:** 2022-08-26

**Authors:** Markie O. Dales, Callum Mitchell, Alison M. Gurney, Robert M. Drummond, Charles Kennedy

**Affiliations:** 1 Strathclyde Institute of Pharmacy & Biomedical Sciences, University of Strathclyde, 161 Cathedral Street, Glasgow, G4 0RE Scotland, UK; 2 Faculty of Life Sciences, University of Manchester, Manchester, M13 9NT UK

**Keywords:** AR-C118925XX, P2Y_2_ receptor, Pulmonary artery, Vasoconstriction, Vasodilation

## Abstract

Pulmonary vascular tone is modulated by nucleotides, but which P2 receptors mediate these actions is largely unclear. The aim of this study, therefore, was to use subtype-selective antagonists to determine the roles of individual P2Y receptor subtypes in nucleotide-evoked pulmonary vasodilation and vasoconstriction. Isometric tension was recorded from rat intrapulmonary artery rings (i.d. 200–500 µm) mounted on a wire myograph. Nucleotides evoked concentration- and endothelium-dependent vasodilation of precontracted tissues, but the concentration–response curves were shallow and did not reach a plateau. The selective P2Y_2_ antagonist, AR-C118925XX, inhibited uridine 5′-triphosphate (UTP)- but not adenosine 5′-triphosphate (ATP)-evoked relaxation, whereas the P2Y_6_ receptor antagonist, MRS2578, had no effect on UTP but inhibited relaxation elicited by uridine 5′-diphosphate (UDP). ATP-evoked relaxations were unaffected by the P2Y_1_ receptor antagonist, MRS2179, which substantially inhibited responses to adenosine 5′-diphosphate (ADP), and by the P2Y_12/13_ receptor antagonist, cangrelor, which potentiated responses to ADP. Both agonists were unaffected by CGS1593, an adenosine receptor antagonist. Finally, AR-C118925XX had no effect on vasoconstriction elicited by UTP or ATP at resting tone, although P2Y_2_ receptor mRNA was extracted from endothelium-denuded tissues using reverse transcription polymerase chain reaction with specific oligonucleotide primers. In conclusion, UTP elicits pulmonary vasodilation via P2Y_2_ receptors, whereas UDP acts at P2Y_6_ and ADP at P2Y_1_ receptors, respectively. How ATP induces vasodilation is unclear, but it does not involve P2Y_1_, P2Y_2_, P2Y_12_, P2Y_13_, or adenosine receptors. UTP- and ATP-evoked vasoconstriction was not mediated by P2Y_2_ receptors. Thus, this study advances our understanding of how nucleotides modulate pulmonary vascular tone.

## Introduction

Adenosine 5′-triphosphate (ATP), uridine 5′-triphosphate (UTP), adenosine 5′-diphosphate (ADP) and uridine 5′-diphosphate (UDP) modulate pulmonary vascular tone in many species, including humans. For example, they all elicited vasoconstriction [1–4] and ATP elicited vasodilation [2, 5] in rat isolated pulmonary vascular bed and arteries. Whilst P2X1 receptors contribute to the contractile action of ATP [6, 7], the roles of individual P2Y receptor subtypes in these actions are less clear.

It is important to know which subtypes are functionally expressed because these nucleotides are endogenous agonists that may contribute to the regulation of pulmonary vascular tone under physiological and pathophysiological conditions [8, 9]. For example, ATP present in and released from red blood cells induces an NO-dependent decrease in pulmonary vascular resistance [10, 11]. Also, extracellular ATP levels are elevated in chronic obstructive pulmonary disease [12], and ADP-induced pulmonary vasodilation is reduced in these patients [13]. In addition, acute hypoxic pulmonary vasoconstriction was inhibited by suramin, a broad spectrum P2X/P2Y receptor antagonist, in perfused rabbit lungs [14] and by blocking P2Y_1_ and P2Y_12_ receptors in pigs in vivo [15]. Thus, targeting purinergic signalling could provide a novel therapeutic approach for treating pulmonary disorders.

A major reason for our poor understanding of the receptors through which nucleotides act is the lack of potent and selective competitive antagonists for most P2X and P2Y receptor subtypes [16–19]. Where these have been developed, they have led to major advances in our understanding of purinergic signalling. For example, selective P2Y_1_ antagonists, such as MRS2179, and P2Y_12_ antagonists, such as ticagrelor, cangrelor, and clopidogrel, made major contributions to the identification of the physiological role of both receptor subtypes in platelet aggregation [20] and of P2Y_1_ receptors in gastrointestinal peristalsis [21]. By using cangrelor, we revealed that P2Y_12_ receptors mediate part of the contractile action of ATP and the entire contraction to ADP in rat pulmonary arteries [7], further demonstrating the usefulness of selective antagonists.

Recently, AR-C118925XX, a potent, selective, and competitive P2Y_2_ receptor antagonist, became available [22–25], and we found that it inhibited UTP-evoked responses in human vascular endothelial cells [26]. The aim of this study, therefore, was to use subtype-selective antagonists, such as AR-C118925XX, to determine the roles of P2Y_2_ receptors and other subtypes in nucleotide-evoked pulmonary vasodilation and vasoconstriction.

## Methods and materials

### Tissue preparation

The methods used conform to the ARRIVE guidelines and meet the ethical requirements of Strathclyde University (https://www.strath.ac.uk/science/biomedicalresearchatstrathclyde/). Segments of rat intrapulmonary artery (rIPA) [3] and rat tail artery (rTA) [27] were prepared for in vitro recording as described previously. Briefly, male Sprague–Dawley rats (200–450 g) were killed by cervical dislocation, according to the schedule 1 guidelines. The heart and lungs were removed en bloc and placed in a cold buffer solution composed of (mM): NaCl 122, KCl 5, N-[2-hydroxyethyl] piperazine-N′-(2-ethanesulfonic acid] (HEPES) 10, KH_2_PO_4_ 0.5, NaH_2_PO_4_ 0.5, MgCl_2_ 1, glucose 11, CaCl_2_ 1.8; titrated to pH 7.3 with NaOH and bubbled with medical air (21% O_2_, 5% CO_2_, 74% N_2_). The rIPA (internal diameter of 300–500 µm) was dissected out, cleaned of connective tissue, and cut into rings ~ 5 mm long. When appropriate, the endothelium was removed by carefully passing a thread or human hair through the lumen. Segments of rTA (internal diameter of 300–500 µm, 5 mm long) were prepared in the same way. Artery rings were mounted horizontally in a static flow, 1 ml organ bath on a pair of intraluminal stainless-steel wires and bathed in the HEPES-based buffer solution, which was continuously bubbled by pumping atmospheric air into the solution (78% N_2_, 21% O_2_, and 0.4% CO_2_). Tissues were equilibrated under a resting tension of 0.5 g (rIPA) or 1 g (rTA) at 37 °C for 60 min and then exposed twice to isotonic 40 mM KCl solution (whole bath replacement) for 5 min, 30 min apart, to verify their integrity. Tissue tension was recorded by Grass FT03 isometric force transducers (Grass Instruments, Quincy, MA) connected to a PowerLab/4e system using Chart 5 software (ADInstruments Ltd., UK). Drugs were added directly to the organ bath and washed out by replacement with a drug-free solution.

### Experimental protocols—vasodilation

Tissues were first contracted with phenylephrine (PE) (0.1 µM) and when the tension had reached a plateau, acetylcholine (ACh) (10 µM) was applied to assess the presence of the endothelium. After washing out the drugs, tension returned to baseline and the tissue was again contracted with PE (0.1 μM) to enable nucleotide-induced vasodilation to be investigated. Initially, concentration–response curves (CRC) to UTP, ATP, UDP, and ADP were generated by the cumulative addition of the agonists. The amplitude of the relaxation evoked by each concentration was calculated as a percentage of the peak amplitude of the PE-evoked contraction. CRC was analysed using nonlinear regression by fitting the Hill equation to the data.

Single agonist concentrations that were on the ascending portions of the CRC were then used in subsequent experiments (3 µM for UTP and UDP and 10 µM for ATP and ADP). Initially, the reproducibility of relaxations evoked in endothelium-intact tissues was determined by adding one of the nucleotides twice, 30 min apart. The role of the endothelium was also investigated in separate tissues. Its physical removal was first confirmed by loss of relaxation to ACh (10 μM), and a nucleotide was then added once. These nucleotide-evoked responses obtained in the absence of endothelium and the second response produced in the presence of endothelium were compared with the first response in the presence of endothelium using one-way ANOVA with Dunnett’s correction.

To investigate the role of individual receptor subtypes, an agonist was applied twice, 30 min apart, to PE-contracted arteries. Once the first control response had been obtained, the tissue was incubated with an antagonist for 20 min before the second addition. For each response, the peak relaxation amplitude was calculated as a percentage of the PE-induced tension and the two values were compared using the Student’s paired *t*-test. Control experiments showed that PE-evoked contractions were reproducible, as when PE was added twice, 30 min apart, the second response was 101.2 ± 2.2% of the first (*n* = 24). Off-target effects of the antagonists on muscle contractility were assessed by comparing the peak amplitude of the PE-evoked contractions in mg in the absence and presence of the antagonist using Student’s paired *t*-test.

### Experimental protocols—vasoconstriction

Nucleotide-evoked concentration-contraction curves in rIPA and rTA do not reach a plateau, so equi-effective concentrations of ATP and UTP (300 μM for rIPA [3, 4] and 1 mM for rTA, [27, 28]), were used and applied at 30 min intervals to arteries at baseline tone. Preliminary experiments found that this protocol elicited reproducible contractions of rIPA when the endothelium was intact but not when it had been removed. Consequently, contractions of rIPA were studied in the presence of the endothelium. In contrast, nucleotide-evoked contractions of rTA were reproducible in both endothelium-intact and -denuded tissues, so to limit the influence of the endothelium, contractions of the rTA were studied in its absence.

To investigate the effect of AR-C118925XX, control contractions to UTP or ATP were obtained. The tissue was then incubated with the antagonist for 20 min before UTP or ATP were reapplied. The peak contraction amplitude was measured and the values obtained in the absence and presence of AR-C118925XX and expressed in mg were compared using the Student’s paired *t*-test.

### P2Y_2_ receptor mRNA expression

The expression of P2Y_2_ receptor mRNA in endothelium-denuded rIPA was investigated as described previously [7]. Briefly, total RNA was prepared from endothelium-denuded IPA of 11 rats (wet tissue weight = 146.6 mg) using a Total RNeasy Midi kit (Qiagen, CA, USA), according to the manufacturer’s protocol. The RNA concentration (42.5 μg/100 μl) was determined spectrophotometrically using a GeneQuant II RNA/DNA calculator (Pharmacia). cDNA was then synthesised using 5 μg RNA and 200 units of SuperScript III reverse transcriptase (Invitrogen) and added to a HotStarTaq DNA polymerase (Qiagen) PCR reaction mix containing 10 pmol/μl of primers designed to selectively recognise P2Y_2_ receptors (forward 5′-GGGACGAACTGGGTTACAAATGTC-3′, reverse 5′-GGTGTGGCAACTGAGGTCAAG-3′) (MWG-Biotech). The mix was placed in a DNA Thermal Cycler (Perkin Elmer, UK), and the following protocol was applied: 10 min at 95 °C followed by 35 cycles of 90 s at 95 °C, 30 s at 52 °C, 90 s at 68 °C, and a final extension step of 10 min at 68 °C. PCR products were separated on a 1.5% *w/v* agarose gel and visualised by ethidium bromide staining. The bands were then purified and the sequence was confirmed using a BigDye v3.1 Terminator Cycle Sequencing Kit (Applied Biosystems, Warrington, UK) and an Applied Biosystems 3100 Avant Genetic Analyser.

### Data analysis

Data are shown as mean ± S.E.M or geometric mean with 95% confidence limits (95% cl) for EC_50_ values. They were analysed statistically as indicated in the protocols above using GraphPad Prism v7.01 (GraphPad Software, San Diego, CA, USA). Differences were considered statistically significant when *P* < 0.05.

### Drugs, materials, and solutions

ATP (Na_2_ salt), ADP (Na salt), UTP (Na_3_(H_2_O)_2_ salt), UDP (Na salt), phenylephrine hydrochloride, and acetylcholine chloride (all Sigma-Aldrich Co, Gillingham, Dorset, UK), MRS2179 (Na_4_ salt) (Tocris, Bristol, UK), cangrelor (a generous gift from The Medicines Company, USA), and suramin hexasodium (RBI, USA) were dissolved in deionised water as 1-, 10-, or 100-mM stock solutions, as appropriate. AR-C118925XX, MRS2578, and CGS15943 (all Tocris, Bristol, UK) were dissolved in DMSO as 10 mM stocks. All were frozen immediately and stored at – 20 °C, then diluted in a buffer on the day of use. Common chemicals were supplied by Sigma-Aldrich Co., Fisher Scientific UK (Loughborough, UK) and VWR International, (Lutterworth, UK) and were of the highest purity available. Isotonic 40 mM K^+^ solution was prepared by replacing NaCl in the HEPES-based buffer solution with an equimolar amount of KCl to maintain the osmolarity of the solution.

## Results

### Nucleotides evoke endothelium-dependent vasodilation at raised tone

**Fig. 1:**
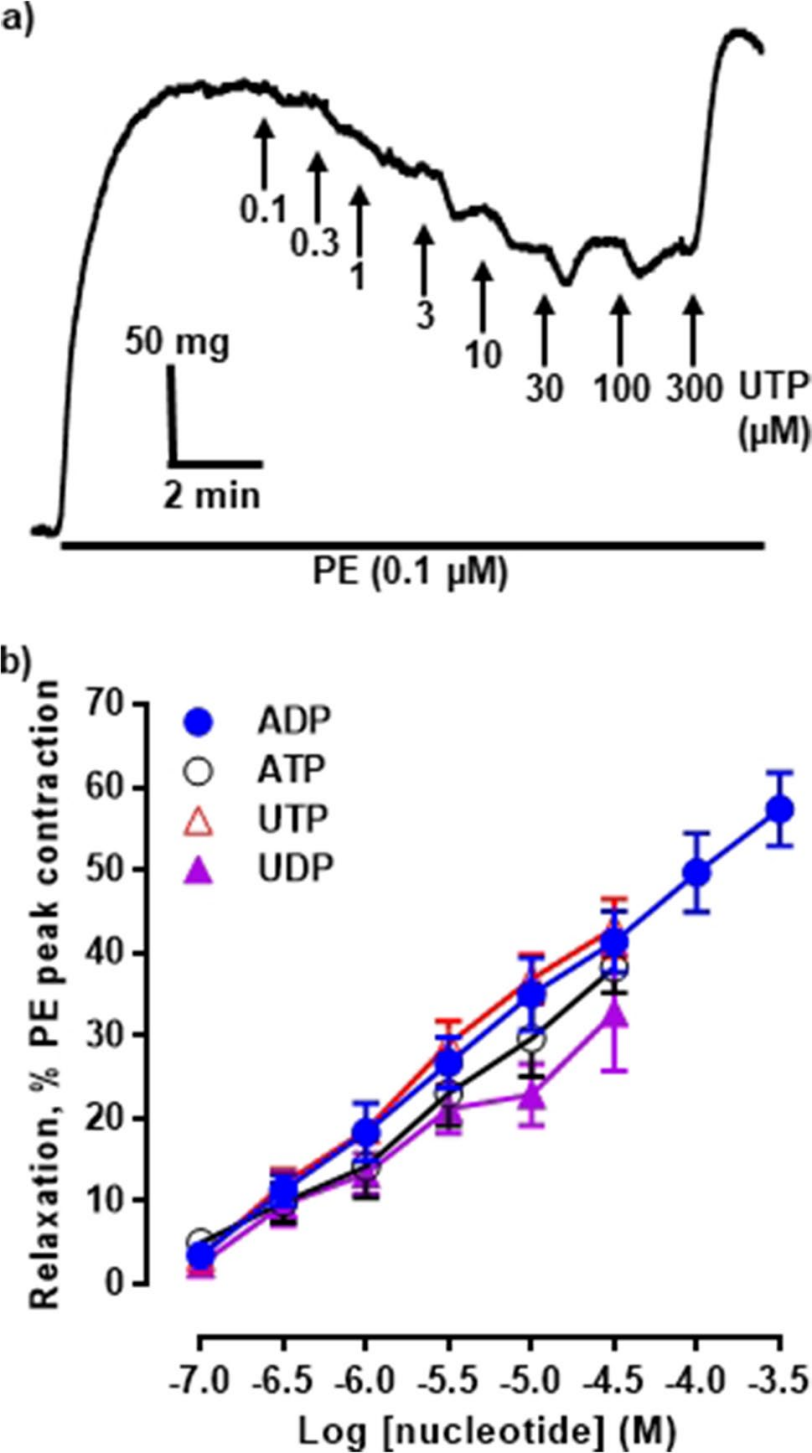
Nucleotides elicit vasodilation of precontracted rIPA. **a** The trace shows typical relaxations of PE (0.1 µM)-precontracted, endothelium-intact rIPA induced by UTP, added cumulatively as indicated by the horizontal bar (PE) and vertical arrows (UTP). **b** The mean peak amplitude of relaxations evoked by cumulative addition of UTP, ATP, UDP, and ADP on PE (0.1 µM) pre-contracted rIPA is shown. Data are expressed as a percentage of the contraction evoked by PE. Vertical lines indicate S.E.M. *n* = 7 UTP, UDP; *n* = 6 ATP, ADP

The aim of the initial experiments was to determine the potencies of the nucleotides in inducing vasodilation of the rIPA by constructing CRC. Following precontraction by PE (0.1 µM), UTP, ATP, UDP (0.1–30 µM), and ADP (0.1–300 µM) elicited concentration-dependent relaxation (Fig. 1a, b). In general, however, the relaxations were not maintained and higher concentrations (30–300 µM) of UTP, ATP, and UDP, but not ADP, tended to evoke biphasic responses, with contractions quickly following the vasodilation. Consequently, a plateau in the CRC was not reached. The goodness of fit (*r*^2^) of the Hill equation to the data was, on the whole, poor, the CRC slopes generated were all shallow and the upper limits for the EC_50_ values of ATP and UDP could not be calculated (not shown). Together, these issues meant that agonist CRC was not a reliable tool with which to characterise the effects of antagonists. Therefore, single concentrations of the nucleotides that were on the ascending portions of the CRC were selected to be used in the subsequent experiments (3 µM for UTP and UDP and 10 µM for ATP and ADP).

**Fig. 2:**
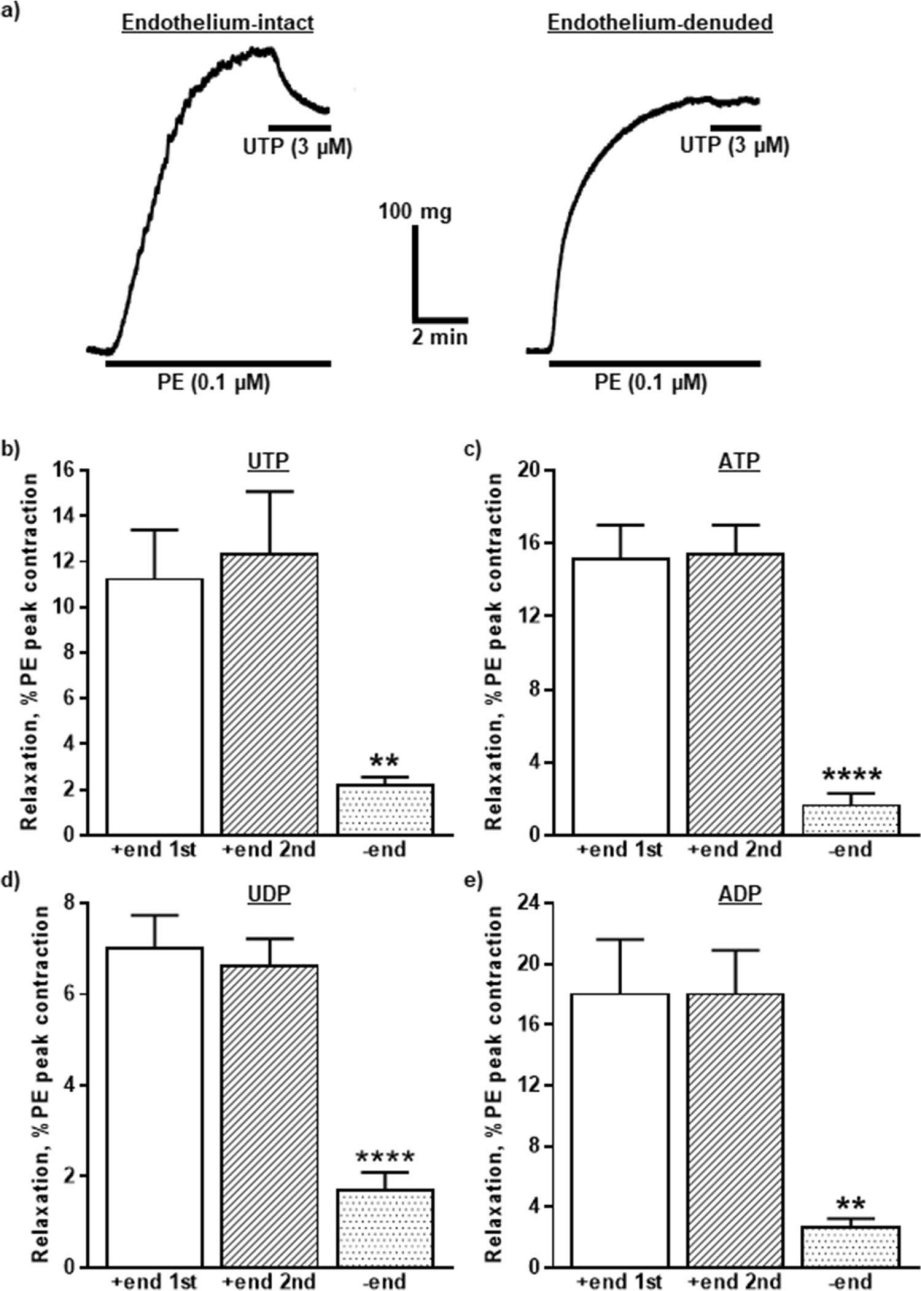
Nucleotide-induced vasodilatation of rIPA is reproducible and endothelium-dependent. **a** The traces show typical relaxations of PE (0.1 µM) pre-contracted rIPA induced by UTP (3 µM) in the presence (left-hand side) and absence (right-hand side) of an intact endothelium. PE and UTP were added as indicated by the horizontal bars. The mean peak amplitude of relaxations evoked by **b** UTP (3 µM), **c** ATP (10 µM), **d** UDP (3 µM), and **e** ADP (10 µM) when added twice to endothelium-intact (+ end) and once to separate endothelium-denuded (− end) tissues is shown. Responses are expressed as a percentage of the contraction evoked by PE. Vertical lines indicate S.E.M. *n* = 6 each. ** *P* < 0.01, **** *P* < 0.0001 for responses in endothelium-denuded rIPA compared to the first response in endothelium-intact Ripa

Under these conditions, the nucleotides evoked relaxations of endothelium-intact rIPA (Fig. 2a) that were reproducible (Fig. 2b–e), as when added twice, 30 min apart, there was no difference in the mean amplitude of the responses, the second being 106.4 ± 6.0% (UTP), 103.9 ± 5.1% (ATP), 95.7 ± 2.8% (UDP), and 103.8 ± 3.6% (ADP) of that of the first (*n* = 6 each). ACh (10 µM) also relaxed these tissues by 86.1 ± 2.1% of the peak amplitude of the contraction evoked by PE (*n* = 24). In endothelium-denuded tissues, the relaxations elicited by the nucleotides were ~ 10–25% of those produced in endothelium-intact tissues (Fig. 2a–e) (*P* < 0.01 for UTP and ADP and *P* < 0.0001 for ATP and UDP) and response to ACh was virtually abolished (1.7 ± 0.7% and *n* = 12, *P* < 0.0001). Thus, nucleotides evoked reproducible relaxations of rIPA that were largely dependent upon an intact endothelial layer.

### Effects of AR-C118925XX on UTP- and ATP-induced vasodilation

**Fig. 3:**
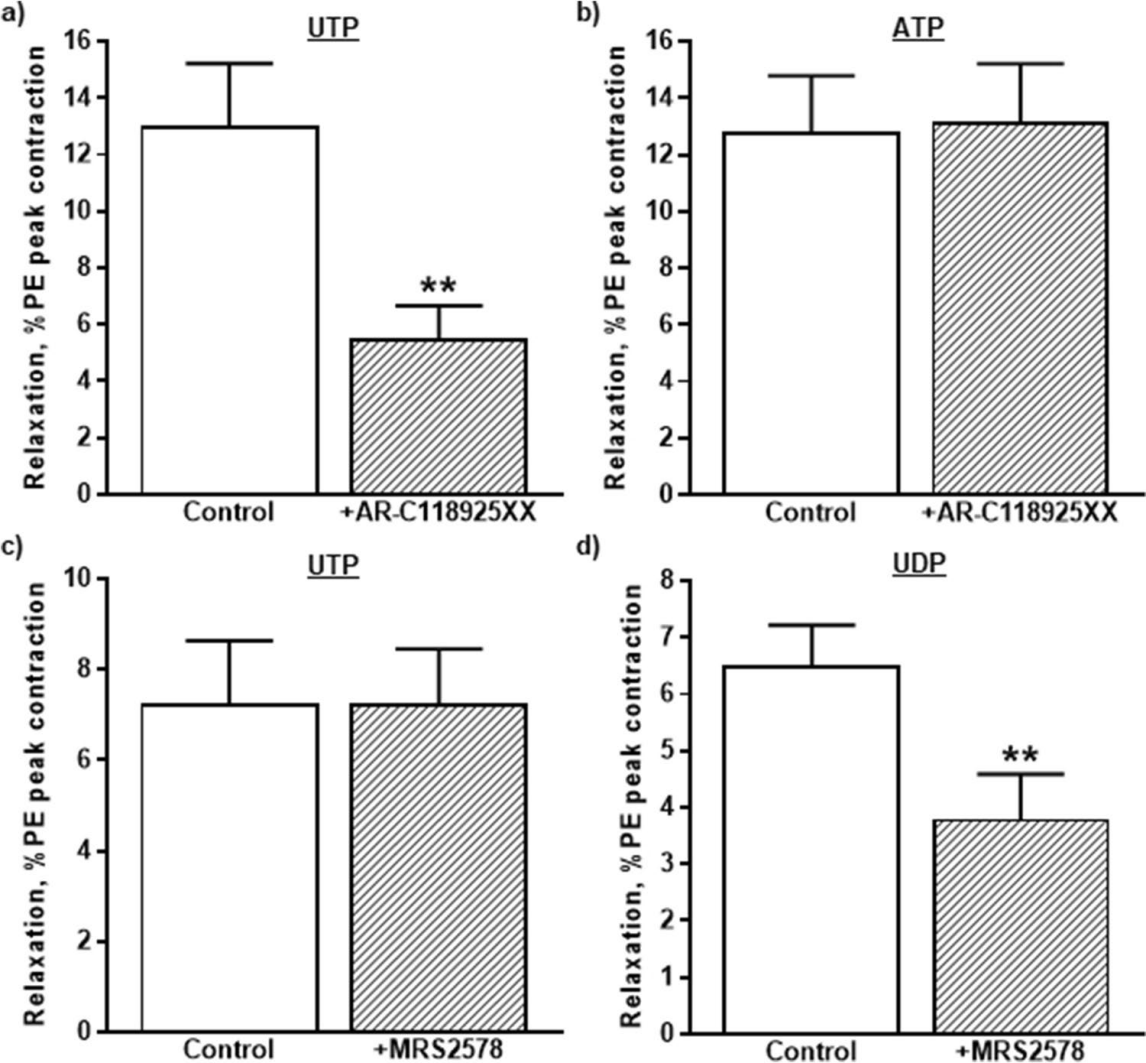
Inhibitory effects of P2Y_2_ and P2Y_6_ receptor antagonists. The mean peak amplitude of the relaxations evoked by **a**, **c** UTP (3 µM), **b** ATP (10 µM), and **d** UDP (3 µM) in the absence and presence of **a**, **b** AR-C118925XX (1 µM) and **c**, **d** MRS2578 (1 µM) are shown. Relaxations are expressed as a percentage of the contraction elicited by PE. Vertical lines indicate S.E.M. *n* = 6 each. ** P < 0.01 for responses to UTP in the presence of AR-C118925XX and to UDP in the presence of MRS2578 compared to the control responses in their absence

Next, the role of P2Y_2_ receptors in the relaxations evoked by UTP and ATP was determined using the selective antagonist, AR-C118925XX, at a concentration (1 µM) that is 270 times greater than its *K*_B_ at P2Y_2_ receptors (3.7 nM) [26]. When preincubated with the tissue for 20 min, AR-C118925XX (1 µM) had no effect on the basal tone of the rIPA or on contractions evoked by PE (0.1 µM) (102.6 ± 2.1% of control, *n* = 12). However, it significantly reduced the vasodilation induced by UTP (3 µM) by 59.2 ± 3.4% (*n* = 6) (Fig. 3a), while having no effect on responses to ATP (10 µM) (102.9 ± 4.3% of control, *n* = 6) (Fig. 3b). Thus, P2Y_2_ receptors contribute substantially to the vasodilatation of the rIPA evoked by UTP, but not ATP.

### Effects of MRS2578 on UTP- and UDP-induced vasodilation

UTP can stimulate P2Y_6_ receptors, although it is much less potent than UDP [29, 30], and may also act indirectly after dephosphorylation by ectonucleotidases to UDP [29]. Therefore, we investigated the effects of the noncompetitive P2Y_6_ receptor antagonist, MRS2578 (1 µM), a concentration that is ten times higher than the IC_50_ (98 nM) obtained at the P2Y_6_ receptor expressed in 1321N1 astrocytoma cells [31]. MRS2578 (1 µM) had no effect on the basal tone of the rIPA, but it slightly inhibited the contractions evoked by PE (0.1 µM) (92.8 ± 1.9% of control, *n* = 12, *P* < 0.05). Whilst MRS2578 had no effect on the relaxations evoked by UTP (3 µM) (102.5 ± 5.0% of control, *n* = 6) (Fig. 3c), it reduced those induced by UDP (3 µM) by 44.8 ± 12.1%, *n* = 6, *P* < 0.01) (Fig. 3d). Thus, UDP, but not UTP, acts at P2Y_6_ receptors to elicit vasodilation of the rIPA.

### Effects of MRS2179 on ATP- and ADP-induced vasodilation

**Fig. 4:**
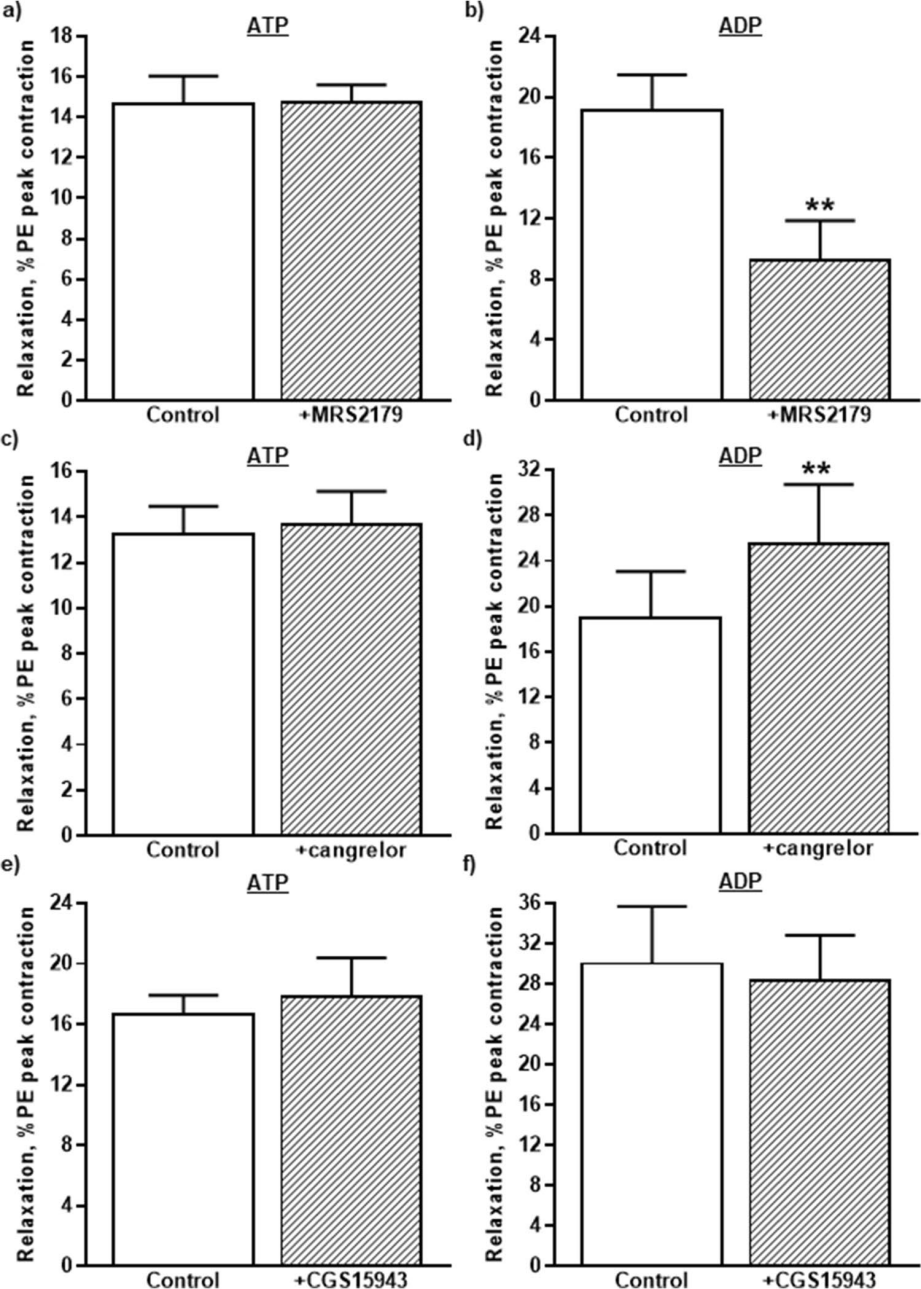
The effects of P2Y_1_, P2Y_12_, and P1 receptor antagonists. The mean peak amplitude of the relaxations evoked by **a**, **c**, **e** ATP (10 µM) and **b**, **d**, **f** ADP (10 µM) in the absence and presence of **a**, **b** MRS2179 (10 µM), **c**, **d** AR-C69913MX (1 µM) or **e**, **f** CGS15943 (1 µM) are shown. Relaxations are expressed as a percentage of the contraction elicited by PE. Vertical lines indicate S.E.M. *n* = 6 each. ** P < 0.01 for responses to ADP in the presence of MRS2179 or AR-C69913MX compared to the control responses in their absence

ATP is a potent P2Y_2_ receptor agonist and the lack of effect of AR-C118925XX on ATP-induced vasodilation was a surprise, so roles for other adenine-nucleotide-sensitive P2Y subtypes were investigated. First, the effects of the selective P2Y_1_ receptor antagonist, MRS2179 (10 µM), which is 100 times higher than its *K*_B_ at P2Y_1_ receptors (100 nM) [32], were determined. MRS2179 (10 µM) had no effect on the basal tone of the rIPA, but it slightly inhibited the contractions evoked by PE (0.1 µM) (92.0 ± 0.9% of control and *n* = 12, *P* < 0.01). It also had no effect on the relaxations produced by ATP (10 µM) (104.5 ± 6.5% of control *n* = 6) (Fig. 4a), but it significantly reduced those evoked by ADP (10 µM) by 53.8 ± 10.0% and *n* = 6, *P* < 0.01) (Fig. 4b). Thus, ADP, but not ATP, acts through P2Y_1_ receptors to induce vasodilation of the rIPA.

### Effects of cangrelor on ATP- and ADP-induced vasodilation

Next, the effects of cangrelor, an antagonist with low/sub-nM potency at P2Y_12_ [33–35] and P2Y_13_ [36, 37] receptors, were investigated. Cangrelor (1 µM) had no effect on the basal tone, the contractions evoked by PE (0.1 µM) (103.3 ± 4.1% of control and *n* = 12) or the relaxations evoked by ATP (10 µM) (104 ± 8.3% of control and *n* = 6) (Fig. 4c). However, it significantly increased the amplitude of the relaxations evoked by ADP (10 µM) by 37.0 ± 6.6% (*n* = 6, *P* < 0.01) (Fig. 4d). This shows that neither ATP nor ADP induce vasodilatation through P2Y_12_ or P2Y_13_ receptors.

### The role of adenosine in ATP- and ADP-induced vasodilation

Finally, extracellular ATP and ADP can be progressively dephosphorylated by ectonucleotidases to adenosine, which can act at P1 receptors to elicit vasodilation [38–40]. The role of this mechanism was investigated using the potent P1 receptor antagonist, CGS15943, at a concentration (1 µM) that substantially blocks P1 receptors [41–43]. CGS15943 (1 µM) had no effect on the basal tone of the rIPA but reduced the contractions evoked by PE (0.1 µM) to 49.2 ± 4.4% of control (*n* = 7 and *P* < 0.0001). In contrast, relaxations evoked by ATP (10 µM) (105.5 ± 8.8% of control and *n* = 4) (Fig. 4e) or ADP (10 µM) (98.4 ± 13.5% of control and *n* = 3) (Fig. 4f) were unaffected, so neither ATP nor ADP act via P1 receptors to induce vasodilation.

### Effects of suramin on vasodilation

**Fig. 5:**
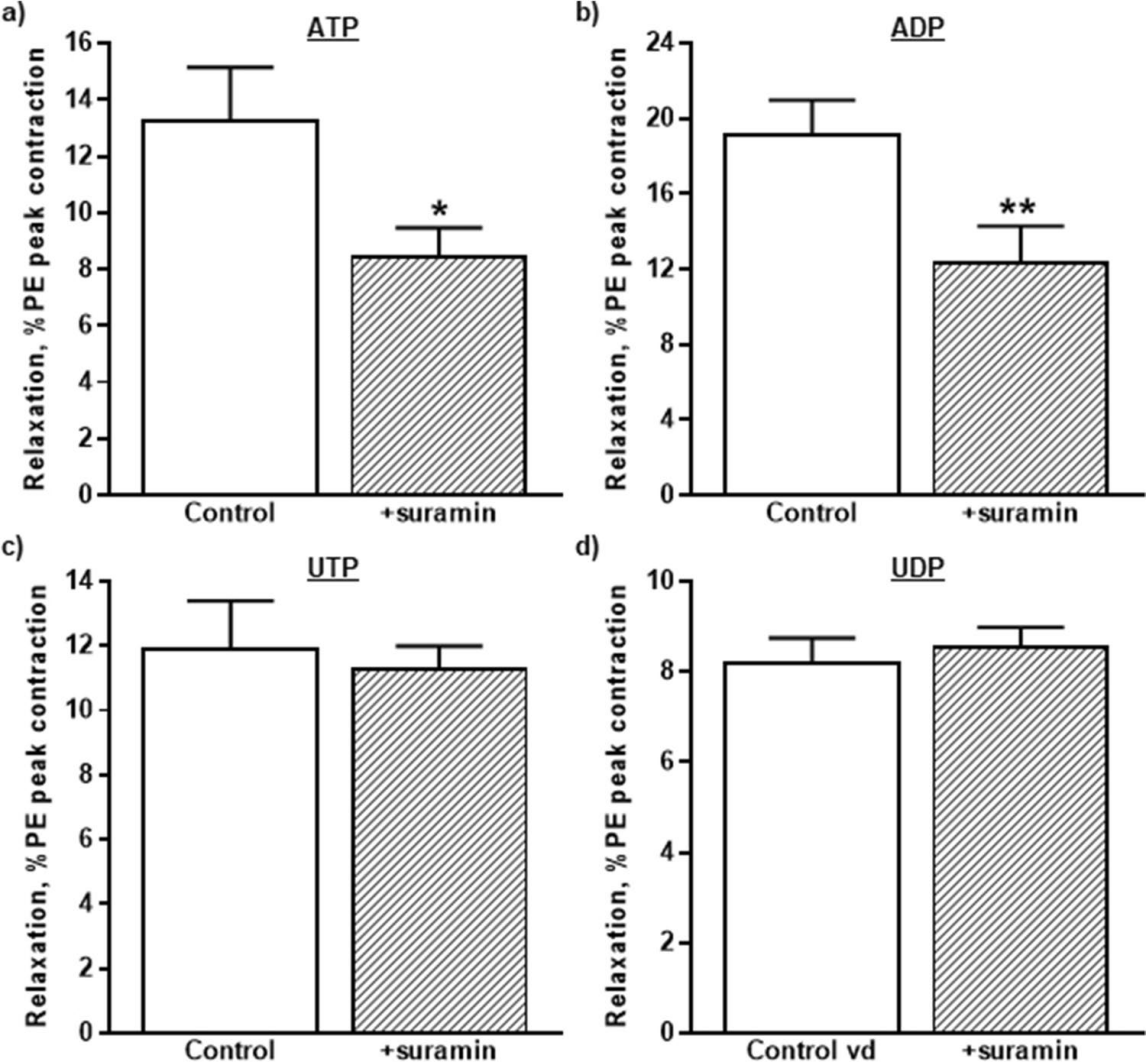
The effects of P2Y_1_, P2Y_12_, and P1 receptor antagonists. The mean peak amplitude of the relaxations evoked by **a** ATP (10 µM), **b** ADP (10 µM), **c** UTP (3 µM), and **d** UDP (3 µM) in the absence and presence of suramin (300 µM) is shown. Relaxations are expressed as a percentage of the contraction elicited by PE. Vertical lines indicate S.E.M. *n* = 6 each. * *P* < 0.05 for responses to ATP and ** *P* < 0.01 for responses to ADP in the presence of suramin compared to the control responses in its absence

4Finally, the nonselective P2 antagonist and suramin [17, 18, 44] were used to further characterise the P2Y receptor subtypes through which the nucleotides might mediate vasodilation of rIPA. Suramin (300 µM) had no effect on the basal tone of the rIPA but reduced the contractions evoked by PE (0.1 µM) to 88.1 ± 2.0% of control (*n* = 24 and *P* < 0.0001). It also significantly reduced the relaxations evoked by ATP (10 µM) by 33.1 ± 8.0% (*n* = 6 and *P* < 0.05) (Fig. 5a) and by ADP (10 µM) by 36.0 ± 7.8% (*n* = 6, *P* < 0.01) (Fig. 5b), but had no effect on responses to UTP (3 µM) (99.5 ± 8.2% of control and *n* = 6) (Fig. 5c) or UDP (3 µM) (105.3 ± 5.7% of control and *n* = 6) (Fig. 5d).

### Effects of AR-C118925XX on UTP- and ATP-induced contractions

**Fig. 6:**
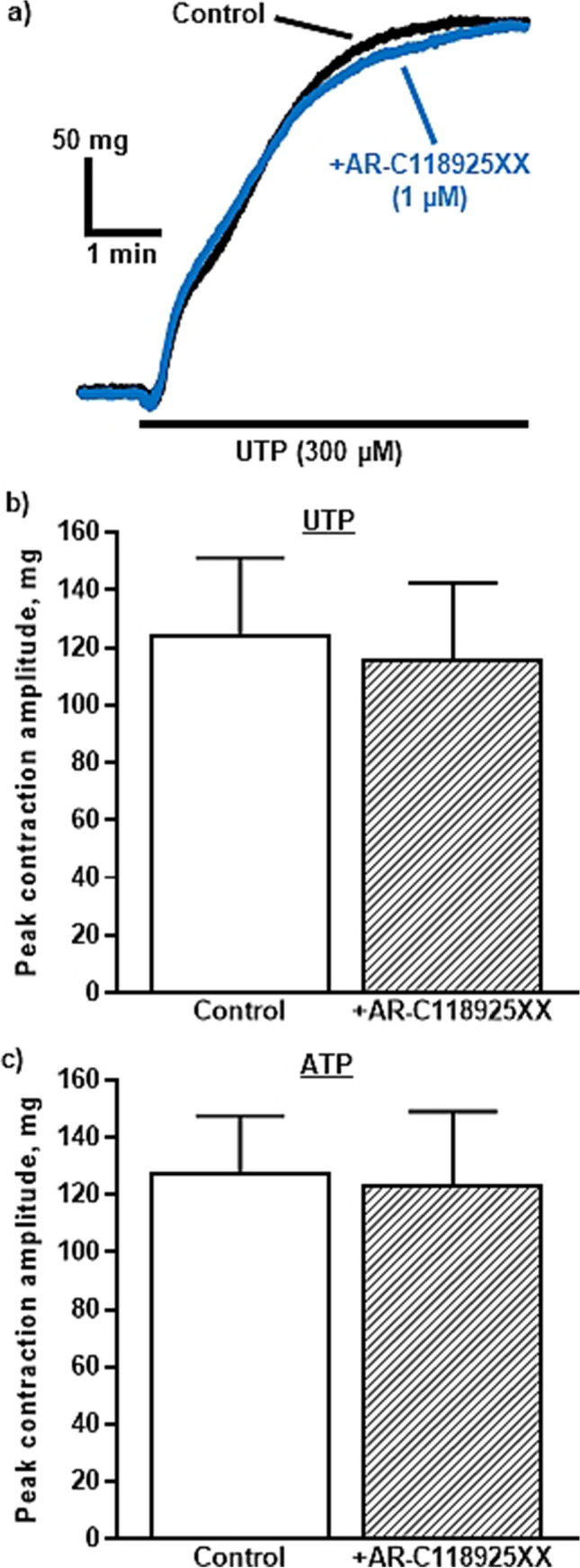
AR-C118925XX has no effect on contractions of the rIPA evoked by UTP and ATP. **a** The superimposed traces show typical contractions of endothelium-intact rIPA evoked by UTP (300 µM) in the absence and presence of AR-C118925XX (1 µM)*.* UTP was added as indicated by the horizontal bar. The mean peak amplitude of contractions evoked by **b** UTP (300 µM) and **c** ATP (300 µM) in the absence and presence of AR-C118925XX (1 µM) is shown. Vertical lines indicate S.E.M. *n* = 6 UTP, *n* = 5 ATP

We previously showed that UTP and ATP contracted the rIPA with moderate potency and that their CRC did not reach a maximum [3, 4]. Therefore, they were applied here at a single, equi-effective concentration (300 µM). Preincubation with AR-C118925XX (1 µM) for 20 min had no effect on the basal tone or on the contractions evoked by UTP (91.8 ± 2.9% control and *n* = 6) (Fig. 6a, b) or ATP (93.5 ± 9.2% control and *n* = 5) (Fig. 6c). Thus, P2Y_2_ receptors do not appear to contribute to contractions of rIPA evoked by UTP and ATP.

**Fig. 7:**
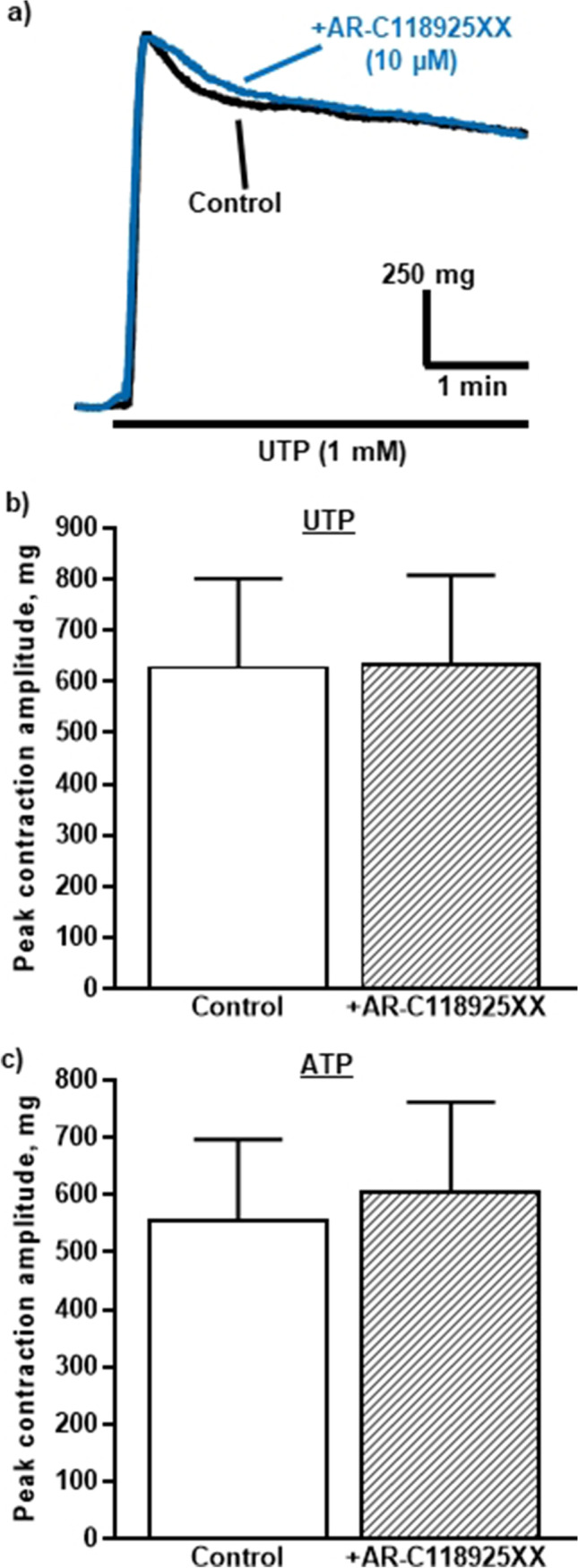
AR-C118925XX has no effect on contractions of the rTA evoked by UTP and ATP. **a** The superimposed traces show typical contractions of endothelium-intact rTA evoked by a UTP (1 mM) in the absence and presence of AR-C118925XX (10 µM)*.* UTP was added as indicated by the horizontal bar. The mean peak amplitude of contractions evoked by **b** UTP (1 mM) and **c** ATP (1 mM) in the absence and presence of AR-C118925XX (10 µM) is shown. Vertical lines indicate S.E.M. *n* = 6 each

This lack of effect of AR-C118925XX was unexpected, so its actions were studied in the rTA, a systemic artery where UTP and ATP also evoke vasoconstriction [27, 28, 45]. In addition, a higher concentration of antagonists was used to produce an even greater level of P2Y_2_ receptor blockade. However, even at 10 µM, AR-C118925XX had no effect on the contractions evoked by UTP (1 mM) (101.3 ± 4.6% of control and *n* = 6) (Fig. 7a, b) or ATP (1 mM) (108.8 ± 9.0% of control and *n* = 6) (Fig. 7c). Thus, P2Y_2_ receptors do not appear to contribute to UTP- or ATP-evoked contractions of the rTA artery either.

### P2Y_2_ receptor expression

**Fig. 8:**
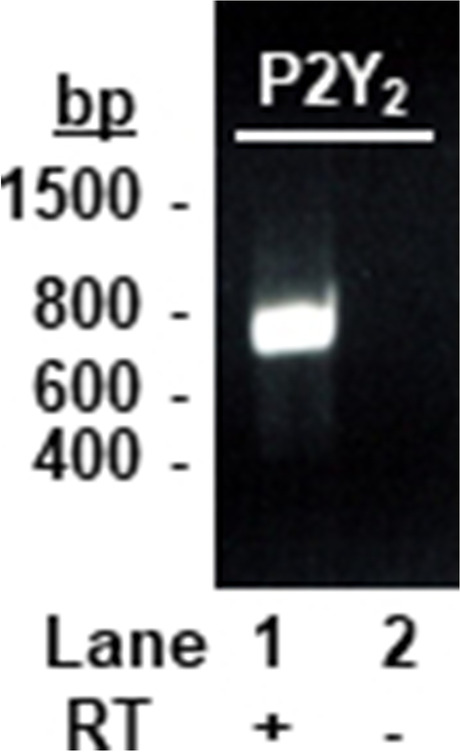
P2Y_2_ receptor mRNA is expressed in the rIPA. Agarose gel electrophoresis of RT-PCR products from endothelium-denuded rIPA using oligonucleotide primers specific for rat P2Y_2_ receptors is shown. This yielded a band in the presence of reverse transcriptase (lane 1), but not in its absence (lane 2). The markers on the left show cDNA band size (base pairs)

We have previously reported the presence of P2Y_1_, P2Y_6_, and P2Y_12_ mRNA in rIPA [7], and here we investigated the expression of P2Y_2_ mRNA. After extracting total RNA from endothelium-denuded rIPA, followed by RT-PCR with specific primers, a single band of the predicted size of the P2Y_2_ receptor was apparent (Fig. 8), and its identity was confirmed by sequencing.

## Discussion

This study showed that adenine and uridine nucleotides elicit endothelium-dependent relaxation of the rIPA. By using the potent and selective antagonist, AR-C118925XX, we found that UTP, but not ATP, acted via P2Y_2_ receptors. Other P2Y subtype-selective antagonists identified P2Y_1_ and P2Y_6_ receptors as sites of action of ADP and UDP, respectively. The mode of action of ATP is unclear but does not involve P2Y_1_, P2Y_2_, P2Y_12_, P2Y_13_, or adenosine receptors. Thus, pulmonary artery endothelium expresses multiple P2Y receptor subtypes, but each nucleotide targets a different receptor subtype to evoke vasodilation. In addition, pulmonary artery P2Y_2_ receptors may be functionally restricted to the endothelium because AR-C118925XX had no effect on contractions evoked by UTP and ATP.

### Vasodilation of rIPA

In the present study, ATP, ADP, UTP, and UDP all evoked concentration-dependent relaxation of rIPA, but the CRC were shallow and did not reach a plateau. This can arise if an agonist acts at more than one receptor and with different potencies to produce its effect and/or if agonist concentration at the biophase next to the plasma membrane is not maintained due to the breakdown of the agonist by ecto-enzymes or its cellular uptake by transporters [46]. Consistent with a role for agonist breakdown, knockout of CD39, an ecto-enzyme that dephosphorylates extracellular tri- and diphosphate nucleotides [47], significantly potentiated contractions of mouse aorta evoked by exogenous UDP and UTP and greatly increased the slope of their CRC [48]. Ideally, the pharmacological profile of an antagonist is determined by generating agonist CRC in its absence and then the presence and quantifying any rightwards shift of the CRC. But these issues meant that this protocol could not be followed and so single concentrations of the nucleotides were used instead.

The nucleotide-evoked relaxations of the rIPA were substantially reduced here upon removal of the endothelium, consistent with previous reports in the rat pulmonary vascular system [2, 5]. Endothelium-dependent vasodilation to ATP and ADP has also been reported in pulmonary arteries obtained from humans [13, 49, 50], ATP in dogs [51], and ATP and UTP in rabbits [52, 53].

### Receptors mediating vasodilation to UTP and UDP

In this study, AR-C118925XX inhibited UTP-evoked relaxation of rIPA by ~ 60%, showing that P2Y_2_ receptors mediate endothelium-dependent vasodilation. This is in line with earlier studies where AR-C118925XX inhibited UTP-induced relaxation of rat carotid artery [54] and associated endothelial signalling events, such as Ca^2+^ mobilisation [26, 55, 56] and eNOS and Akt phosphorylation [57]. Similarly, P2Y_2_ receptor knockdown reduced UTP-evoked Ca^2+^ mobilisation in human endothelial cells [58]. The consequence of P2Y_2_ receptor knockout is, however, more complicated, as it had no effect on endothelium-dependent vasodilation of mouse aorta induced by UTP [59] but abolished the relaxation of mouse coronary artery evoked by the selective P2Y_2_ agonist UTPγS [60]. A possible explanation is that in the absence of P2Y_2_ receptors, UTP can act at other P2Y subtypes to elicit vasodilation.

The AR-C118925XX-resistant component of UTP-evoked vasodilation in rIPA was not mediated by P2Y_6_ receptors, as the P2Y_6_ antagonist, MRS2578, had no effect, even though it inhibited UDP by ~ 45%. This result also indicates that dephosphorylation of UTP by ectonucleotidases to produce UDP does not contribute to its action. It is likely that P2Y_6_ receptors play an even greater role in the action of UDP, as the concentration of MRS2578 used, 1 µM, is submaximal [31]. Higher concentrations were not applied because they can act at sites other than P2Y_6_ receptors [7].

Unexpectedly, the nonselective antagonist, suramin, was ineffective against UTP, even though it is a P2Y_2_ receptor antagonist [61]. P2Y_4_ receptors are suramin insensitive [61], so they could, in theory play a role in the antagonist-resistant components of vasodilation, but a selective P2Y_4_ receptor antagonist is not currently available. Note, however, that P2Y_4_ receptor knockout had no effect on the endothelium-dependent relaxation of mouse aorta evoked by UTP or UDP [62]. Nonetheless, the present experiments identify P2Y_2_ and P2Y_6_ receptors as mediators of endothelium-dependent vasodilation in rIPA.

### Receptors mediating vasodilation to ATP and ADP

In contrast to the inhibition of UTP, AR-C118925XX had no effect in this study on the relaxation of the rIPA evoked by ATP. This was unexpected, as ATP is a potent P2Y_2_ receptor agonist [16, 18] and P2Y_2_ receptor knockout shifted the relaxant CRC for ATP in the mouse aorta rightwards and decreased the maximum response [59]. ATP is a partial agonist at P2Y_1_ receptors [63], but this subtype was not involved, as the P2Y_1_ antagonist, MRS2179, was also ineffective against ATP. This result also indicates that dephosphorylation of ATP by ectonucleotidases does not produce enough ADP to stimulate P2Y_1_ receptors here. MRS2179 also had no effect on ATP-induced, endothelium-dependent vasodilation of the rat mesenteric bed [64] or mouse aorta [62]. It is of interest to note, however, that the contribution of P2Y_1_ receptors to the action of ATP in the aorta was increased in P2Y_2_ knockout mice [59], indicating that it can play a role under some conditions. Finally, the lack of inhibition by CGS1593 shows that adenosine receptors do not contribute to relaxation evoked by adenine nucleotides.

Unlike the other antagonists, suramin did, however, suppress the ATP-induced relaxation of the rIPA, confirming a role for P2 receptors, but its site of action is unclear. Of the other ATP-sensitive P2Y subtypes, the P2Y_4_ receptor is suramin-insensitive and the P2Y_11_ receptor, although suramin-sensitive, is not expressed in rats [65]. Most P2X receptor subtypes are not expressed in endothelial cells [66] and although there is strong evidence that endothelial P2X4 receptors mediate vasodilation [67–69], this subtype has, at most, very low sensitivity to suramin [19]. Thus, at present, the receptor(s) through which ATP acts to cause vasodilation of the rIPA remains uncertain. It is now clear that G protein-coupled receptors, including P2Y receptors [70], can interact to form dimeric or higher-ordered oligomeric complexes with novel pharmacological and signalling properties, so this might underlie the pharmacological profile of ATP’s action in rIPA.

ADP-evoked relaxation of the rIPA was substantially inhibited by MRS2179 and suramin, both of which block P2Y_1_ receptors [16, 18]. ADP also stimulates P2Y_12_ and P2Y_13_ receptors, but the P2Y_12/13_ receptor antagonist, cangrelor, potentiated rather than inhibited ADP-induced vasodilation. This is likely due to inhibition of the counteractive vasoconstriction induced by ADP via smooth muscle P2Y_12/13_ receptors [7]. Like ATP, ADP was unaffected by CGS1593. Together, these data indicate that P2Y_1_ receptors are the major site of relaxation of rIPA by ADP and that P2Y_12/13_ and adenosine receptors are not involved.

### Modulation of PE-induced vasoconstriction by purinergic antagonists

In this study, several of the antagonists reduced the vasoconstriction induced by PE, indicating that they may have off-target inhibitory effects. This is consistent with a previous report that MRS2578 reduced KCl-evoked contractions of rIPA [7]. On the other hand, endogenous ATP, released from vascular smooth muscle cells via pannexin-1 channels, has been shown to contribute to α_1_-receptor-mediated vasoconstriction [71–74]. Therefore, the inhibitory effect of suramin against PE may be due to it inhibiting the contractile action of released ATP, most likely at P2X1 receptors. In contrast, the mechanisms underlying the large inhibitory action of CGS15943 on PE-evoked contractions and also the small suppression by MRS2179, are unclear.

### Vasoconstriction of rIPA

In the present study, a high concentration of AR-C118925XX (1 µM) had no effect on UTP- or ATP-evoked contractions of rIPA, indicating no role for P2Y_2_ receptors. Consistent with this, knocking out P2Y_2_ receptors had no effect on UTP-evoked contractions of mouse coronary arteries [60]. Interestingly, a tenfold higher concentration of AR-C118925XX virtually abolished contractions of rat small pulmonary veins induced by ATP [75]. The inactivity of AR-C118925XX in the rIPA is unlikely to be because the concentration used was too low, as it did inhibit UTP-evoked vasodilation. In addition, 10 µM AR-C118925XX had no effect here on UTP- or ATP-evoked contractions of the rTA.

The lack of effect of AR-C118925XX against ATP in the rIPA supports our earlier data, which showed that ATP induced contraction via P2X1 and P2Y_12_ receptors [7]. In contrast, the site of action of UTP is unclear. We previously reported that suramin reduced UTP-evoked contractions, suggesting a role for P2Y_2_ receptors [3]. Consistent with this, we found P2Y_2_ mRNA in the endothelium-denuded rIPA. The lack of effect of AR-C118925XX indicates, however, that other P2Y subtypes must be involved. P2Y_14_ receptors are unlikely to contribute, as the P2Y_14_ agonist, UDP-glucose, did not contract the rIPA [7]. UTP-evoked contractions of the mouse aorta were unaffected by the knockout of P2Y_4_ receptors and abolished by P2Y_6_ knockout [48], but pulmonary artery responses were not examined. Heteromeric receptor complexes with novel pharmacological properties might be involved [70].

### Conclusion

This study showed that UTP, UDP, ATP, and ADP all evoked endothelium-dependent vasodilation of the rIPA. UTP acted predominantly at P2Y_2_ receptors, whereas UDP acted via P2Y_6_ receptors and ADP mainly via P2Y_1_ receptors. The site of action of ATP is unclear but does not involve P2Y_1_, P2Y_2_, P2Y_12_, P2Y_13_, or adenosine receptors. UTP and ATP also produced vasoconstriction, which was unaffected by AR-C118925XX, indicating that contractile P2Y_2_ receptors are not functionally expressed in rIPA vascular smooth muscle. Together, these data represent the most detailed pharmacological characterisation of the receptors that mediate endothelium-dependent vasodilation of pulmonary arteries and extend our understanding of the contractile purinergic receptors.

## Data Availability

The datasets generated and analysed during the current study are available from the corresponding author on reasonable request.

## Abbreviations

95% cl: 95% Confidence limits
ACh: Acetylcholine
ADP: Adenosine 5′-diphosphate
ATP: Adenosine 5′-triphosphate
CRC: Concentration-response curves
PE: Phenylephrine
rIPA: Rat isolated intrapulmonary artery
rTA: Rat isolated tail artery
UDP: Uridine 5′-diphosphate
UTP: Uridine 5′-triphosphate

## References

[1] Rubino A, Burnstock G (1996). Evidence for a P2-purinoceptor mediating vasoconstriction by UTP, ATP and related nucleotides in the isolated pulmonary vascular bed of the rat. Br J Pharmacol.

[2] Rubino A, Ziabary L, Burnstock G (1999). Regulation of vascular tone by UTP and UDP in isolated intrapulmonary arteries. Eur J Pharmacol.

[3] Chootip K, Ness K, Wang Y, Gurney AM, Kennedy C (2002). Regional variation in P2 receptor expression in the rat pulmonary arterial circulation. Br J Pharmacol.

[4] Chootip K, Gurney AM, Kennedy C (2005). Multiple P2Y receptors couple to calcium-dependent, chloride channels in smooth muscle cells of the rat pulmonary artery. Resp Res.

[5] Hasséssian H, Burnstock G (1995). Interacting roles of nitric oxide and ATP in the pulmonary circulation of the rat. Br J Pharmacol.

[6] Syed NH, Tengah A, Paul A, Kennedy C (2010). Characterisation of P2X receptors expressed in rat pulmonary arteries. Eur J Pharmacol.

[7] Mitchell C, Syed NH, Tengah A, Gurney AM, Kennedy C (2012). Identification of contractile P2Y_1_, P2Y_6_ and P2Y_12_ receptors in rat intrapulmonary artery using selective ligands. J Pharmacol Exp Therap.

[8] Cai Z, Guignabert C, Merkus D, Zhou Z (2020). Purinergic dysfunction in pulmonary arterial hypertension. J Am Heart Assoc.

[9] Strassheim D, Verin A, Batori R, Nijmeh H, Burns N, Kovacs-Kasa A, Umapathy NS, Kotamarthi J, Gokhale YS, Karoor V, Stenmark KR, Gerasimovskaya E (2020). P2Y purinergic receptors, endothelial dysfunction, and cardiovascular diseases. Int J Mol Sci.

[10] Sprague RS, Ellsworth ML, Stephenson AH, Lonigro AJ (1996). ATP: the red blood cell link to NO and local control of the pulmonary circulation. Am J Physiol.

[11] Sprague RS, Olearczyk JJ, Spence DM, Stephenson AH, Sprung RW, Lonigro AJ (2003). Extracellular ATP signaling in the rabbit lung: erythrocytes as determinants of vascular resistance. Am J Physiol.

[12] Lommatzsch M, Cicko S, Müller T, Lucattelli M, Bratke K, Stoll P, Grimm M, Dürk T, Zissel G, Ferrari D, Di Virgilio F, Sorichter S, Lungarella G, Virchow JC, Idzko M (2010). Extracellular adenosine triphosphate and chronic obstructive pulmonary disease. Am J Resp Crit Care Med.

[13] Dinh-Xuan AT, Higenbottam TW, Clelland CA, Pepke-Zaba J, Cremona G, Butt AY, Large SR, Wells FC, Wallwork J (1991). Impairment of endothelium-dependent pulmonary-artery relaxation in chronic obstructive lung disease. N Eng J Med.

[14] Baek EB, Yoo HY, Park SJ, Kim HS, Kim SD, Earm YE, Kim SJ (2008). Luminal ATP-induced contraction of rabbit pulmonary arteries and role of purinoceptors in the regulation of pulmonary arterial pressure. Pflugers Arch.

[15] Kylhammar D, Bune LT, Rådegran G (2014). P2Y_1_ and P2Y_12_ receptors in hypoxia- and adenosine diphosphate-induced pulmonary vasoconstriction *in vivo* in the pig. Eur J Appl Physiol.

[16] Jacobson KA, Delicado EG, Gachet C, Kennedy C, von Kügelgen I (2020). Update of P2Y receptor pharmacology : IUPHAR review 27. Br J Pharmacol.

[17] Alexander SP, Mathie A, Peters JA, Veale EL, Striessnig J, Kelly E (2021). the concise guide to pharmacology 2021/22: ion channels. Br J Pharmacol.

[18] Alexander SP, Christopoulos A, Davenport AP, Kelly E, Mathie A, Peters JA, Veale EL (2021). The concise guide to pharmacology 2021/22: G protein-coupled receptors. Br J Pharmacol.

[19] Illes P, Müller CE, Jacobson KA, Grutter T, Nicke A, Fountain SJ, Kennedy C (2021). Update of P2X receptor properties and their pharmacology; IUPHAR review. Br J Pharmacol.

[20] Abbracchio MP, Burnstock G, Boeynaems JM, Barnard EA, Boyer JL, Kennedy C, Fumagalli M, Gachet C, Jacobson KA, Weisman GA (2006). International Union of Pharmacology. Update of the P2Y G protein-coupled nucleotide receptors: from molecular mechanisms and pathophysiology to therapy. Pharmacol Rev.

[21] Kennedy C (2015). ATP as a cotransmitter in the autonomic nervous system. Auton Neurosci Basic Clin.

[22] Kindon N, Davis A, Dougall I, Dixon J, Johnson T, Walters I, Thom S, McKechnie K, Meghani P, Stocks MJ (2017). From UTP to AR-C118925, the discovery of a potent non-nucleotide antagonist of the P2Y_2_ receptor. Bioorg Med Chem Lett.

[23] Rafehi M, Burbiel JC, Attah IY, Abdelrahman A, Müller CE (2017). Synthesis, characterization, and *in vitro* evaluation of the selective P2Y_2_ receptor antagonist AR-C118925. Purinergic Signal.

[24] Rafehi M, Neumann A, Baqi Y, Malik EM, Wiese M, Namasivayam V, Müller CE (2017). Molecular recognition of agonists and antagonists by the nucleotide-activated G protein-coupled P2Y_2_ receptor. J Med Chem.

[25] Rafehi M, Müller CE (2018). Tools and drugs for uracil nucleotide-activated P2Y receptors. Pharmacol Ther.

[26] Muoboghare MO, Drummond R, Kennedy C (2019). Characterisation of P2Y_2_ receptors in human vascular endothelial cells using AR-C118925XX, a potent and selective P2Y_2_ antagonist. Br J Pharmacol.

[27] Evans RJ, Kennedy C (1994). Characterisation of P_2_-purinoceptors in the smooth muscle of the rat tail artery; a comparison between contractile and electrophysiological responses. Br J Pharmacol.

[28] McLaren GJ, Buchanan KJ, Burke KS, Sneddon P, Kennedy C (1998). Evidence that ATP acts at two sites to evoke contraction in the rat isolated tail artery. Br J Pharmacol.

[29] Nicholas RA, Watt WC, Lazarowski ER, Li Q, Harden K (1996). Uridine nucleotide selectivity of three phospholipase C-activating P 2 receptors: Identification of a UDP-selective, a UTP-selective, and an ATP- and UTP-specific receptor. Mol Pharmacol.

[30] Filippov AK, Webb TE, Barnard EA, Brown DA (1999). Dual coupling of heterologously-expressed rat P2Y_6_ nucleotide receptors to N-type Ca^2+^ and M-type K^+^ currents in rat sympathetic neurones. Br J Pharmacol.

[31] Mamedova LK, Joshi BV, Gao ZG, Von Kügelgen I, Jacobson KA (2004). Diisothiocyanate derivatives as potent, insurmountable antagonists of P2Y_6_ nucleotide receptors. Biochem Pharmacol.

[32] Boyer JL, Mohanram A, Camaioni E, Jacobson KA, Harden TK (1998). Competitive and selective antagonism of P2Y_1_ receptors by N^6^-methyl 2'-deoxyadenosine 3',5'-bisphosphate. Br J Pharmacol.

[33] Ingall AH, Dixon J, Bailey A, Coombs ME, Cox D (1999). Antagonists of the platelet P2T receptor: a novel approach to antithrombotic therapy. J Med Chem.

[34] Takasaki J, Kamohara M, Saito T, Matsumoto M, Matsumoto S, Ohishi T, Soga T, Matsushime H, Furuichi K (2001). Molecular cloning of the platelet P2T_AC_ ADP receptor: pharmacological comparison with another ADP receptor, the P2Y_1_ receptor. Mol Pharmacol.

[35] Hoffmann K, Sixel U, Di Pasquale F, von Kügelgen I (2008). Involvement of basic amino acid residues in transmembrane regions 6 and 7 in agonist and antagonist recognition of the human platelet P2Y_12_-receptor. Biochem Pharmacol.

[36] Marteau F, Le Poul E, Communi D, Communi D, Labouret C, Savi P, Boeynaems JM, Gonzalez NS (2003). Pharmacological characterization of the human P2Y_13_ receptor. Mol Pharmacol.

[37] Fumagalli M, Trincavelli L, Lecca D, Martini C, Ciana P, Abbracchio MP (2004). Cloning, pharmacological characterisation and distribution of the rat G-protein-coupled P2Y_13_ receptor. Biochem Pharmacol.

[38] Burnstock G, Kennedy C (1986). A dual function for adenosine triphosphate in the regulation of vascular tone: excitatory cotransmitter with noradrenaline from perivascular nerves and locally released inhibitory intravascular agent. Circ Res.

[39] Rayment SJ, Ralevic V, Barrett DA, Cordell R, Alexander SP (2007). A novel mechanism of vasoregulation: ADP-induced relaxation of the porcine isolated coronary artery is mediated via adenosine release. FASEB J.

[40] Alsaqati M, Chan SL, Ralevic V (2014). Investigation of the functional expression of purine and pyrimidine receptors in porcine isolated pancreatic arteries. Purinergic Signal.

[41] Ongini E, Dionisotti S, Gessi S, Irenius E, Fredholm BB (1999). Comparison of CGS 15943, ZM 241385 and SCH 58261 as antagonists at human adenosine receptors. Naunyn-Schmiedeberg’s Arch Pharmacol.

[42] Ilie A, Raimondo JV, Akerman CJ (2012). Adenosine release during seizures attenuates GABA A receptor-mediated depolarization. J Neurosci.

[43] Sivaramakrishnan V, Bidula S, Campwala H, Katikaneni D, Fountain SJ (2012). Constitutive lysosome exocytosis releases ATP and engages P2Y receptors in human monocytes. J Cell Sci.

[44] Kennedy C, Chootip K, Mitchell C, Syed NH, Tengah A (2013). P2X and P2Y nucleotide receptors as targets in cardiovascular disease. Future Med Chem.

[45] Tengah A, Syed NH, Abdul Talip ST, Bujang SNB, Kennedy C (2018). Comparison of signalling mechanisms underlying UTP-evoked vasoconstriction of rat pulmonary and tail arteries. Eur J Pharmacol.

[46] Kennedy C, Leff P (1995). How should P_2X_-purinoceptors be characterised pharmacologically?. Trends Pharmacol Sci.

[47] Robson SC, Sévigny J, Zimmermann H (2006). The E-NTPDase family of ectonucleotidases: structure function relationships and pathophysiological significance. Purinergic Signal.

[48] Kauffenstein G, Drouin A, Thorin-Trescases N, Bachelard H, Robaye B, D'Orléans-Juste P, Marceau F, Thorin E, Sévigny J (2010). NTPDase1 (CD39) controls nucleotide-dependent vasoconstriction in mouse. Cardiovasc Res.

[49] Greenberg B, Rhoden K, Barnes PJ (1987). Endothelium-dependent relaxation of human pulmonary arteries. Am J Physiol.

[50] Liu SF, McCormack DG, Evans TW, Barnes PJ (1989). Characterization and distribution of P2-purinoceptor subtypes in rat pulmonary vessels. J Pharmacol Exp Ther.

[51] De Mey JG, Vanhoutte PM (1982). Heterogeneous behavior of the canine arterial and venous wall. Importance Endothel Circ Res.

[52] Qasabian RA, Schyvens C, Owe-Young R, Killen JP, Macdonald PS, Conigrave AD, Williamson DJ (1997). Characterization of the P2 receptors in rabbit pulmonary artery. Br J Pharmacol.

[53] Konduri GG, Bakhutashvili I, Frenn R, Chandrasekhar I, Jacobs ER, Khanna AK (2004). P2Y purine receptor responses and expression in the pulmonary circulation of juvenile rabbits. Am J Physiol.

[54] Matsumoto T, Kojima M, Takayanagi K, Katome T, Taguchi K, Kobayashi T (2020). Impaired UTP-induced relaxation in the carotid arteries of spontaneously hypertensive rats. Purinergic Signal.

[55] Lee MD, Wilson C, Saunter CD, Kennedy C, Girkin JM, McCarron JG (2018). Spatially-structured cell populations process multiple sensory signals in parallel in intact vascular endothelium. Sci Signal.

[56] Leong IL, Tsai TY, Wong KL, Shiao LR, Cheng KS, Chan P, Leung YM (2018). Valproic acid inhibits ATP-triggered Ca^2+^ release via a p38-dependent mechanism in bEND.3 endothelial cells. Fund Clin Pharmacol.

[57] Wang S, Iring A, Strilic B, Albarrán Juárez J, Kaur H, Troidl K, Tonack S, Burbiel JC, Müller CE, Fleming I, Lundberg JO, Wettschureck N, Offermanns S (2015). P2Y_2_ and Gq/G_11_ control blood pressure by mediating endothelial mechanotransduction. J Clin Invest.

[58] Raqeeb A, Sheng J, Ao N, Braun AP (2011). Purinergic P2Y_2_ receptors mediate rapid Ca^2+^ mobilization, membrane hyperpolarization and nitric oxide production in human vascular endothelial cells. Cell Calcium.

[59] Guns PJ, Van Assche T, Fransen P, Robaye B, Boeynaems JM, Bult H (2006). Endothelium-dependent relaxation evoked by ATP and UTP in the aorta of P2Y_2_-deficient mice. Br J Pharmacol.

[60] Haanes KA, Spray S, Syberg S, Jørgensen NR, Robaye B, Boeynaems JM, Edvinsson L (2016). New insights on pyrimidine signalling within the arterial vasculature - different roles for P2Y_2_ and P2Y_6_ receptors in large and small coronary arteries of the mouse. J Mol Cell Cardiol.

[61] Charlton SJ, Brown CA, Weisman GA, Turner JT, Erb L, Boarder MR (1996). PPADS and suramin as antagonists at cloned P2Y- and P2U-purinoceptors. Br J Pharmacol.

[62] Guns PJ, Korda A, Crauwels HM, Van Assche T, Robaye B, Boeynaems JM, Bult H (2005). Pharmacological characterization of nucleotide P2Y receptors on endothelial cells of the mouse aorta. Br J Pharmacol.

[63] Palmer RK, Boyer JL, Schachter JB, Nicholas RA, Harden TK (1998). Agonist action of adenosine triphosphates at the human P2Y_1_ receptor. Mol Pharmacol.

[64] Buvinic S, Briones R, Huidobro-Toro JP (2002). P2Y_1_ and P2Y_2_ receptors are coupled to the NO/cGMP pathway to vasodilate the rat arterial mesenteric bed. Br J Pharmacol.

[65] Kennedy C (2017). P2Y_11_ receptors: properties, distribution and functions. Adv Exp Med Biol: Prot Rev.

[66] Burnstock G, Knight GE (2004). Cellular distribution and functions of P2 receptor subtypes in different systems. Int Rev Cytol.

[67] Bodin P, Bailey D, Burnstock G (1991). Increased flow-induced ATP release from isolated vascular endothelial cells but not smooth muscle cells. Br J Pharmacol.

[68] Yamamoto K, Korenaga R, Kamiya A, Ando J (2000). Fluid shear stress activates Ca^2+^ influx into human endothelial cells via P2X4 purinoceptors. Circ Res.

[69] Yamamoto K, Sokabe T, Matsumoto T, Yoshimura K, Shibata M (2006). Impaired flow-dependent control of vascular tone and remodeling in P2X4-deficient mice. Nature Med.

[70] Kennedy C (2021). That was then, this is now: the development of our knowledge and understanding of P2 receptor subtypes. Purinergic Signal.

[71] Billaud M, Lohman AW, Straub AC, Looft-Wilson R, Johnstone SR, Araj CA, Best AK, Chekeni FB, Ravichandran KS, Penuela S, Laird DW, Isakson BE (2011). Pannexin1 regulates α_1_-adrenergic receptor-mediated vasoconstriction. Circ Res.

[72] Billaud M, Chiu YH, Lohman AW, Parpaite T, Butcher JT (2015). A molecular signature in the pannexin1 intracellular loop confers channel activation by the α_1_ adrenoreceptor in smooth muscle cells. Sci Signal.

[73] Kauffenstein G, Tamareille S, Prunier F, Roy C, Ayer A (2016). Central role of P2Y_6_ UDP receptor in arteriolar myogenic tone. Arterioscler Thromb Vasc Biol.

[74] Begandt D, Good ME, Keller AS, DeLalio LJ, Rowley C, Isakson BE, Figueroa XF (2017). Pannexin channel and connexin hemichannel expression in vascular function and inflammation. BMC Cell Biol.

[75] Henriquez M, Fonseca M, Perez-Zoghbi JF (2018). Purinergic receptor stimulation induces calcium oscillations and smooth muscle contraction in small pulmonary veins. J Physiol.

